# Polerovirus genomic variation

**DOI:** 10.1093/ve/veab102

**Published:** 2021-12-04

**Authors:** Katherine LaTourrette, Natalie M Holste, Hernan Garcia-Ruiz

**Affiliations:** Nebraska Center for Virology, University of Nebraska-Lincoln, 4240 Fair Street, Lincoln, NE 68583, USA; Department of Plant Pathology, University of Nebraska-Lincoln, 406 Plant Science Hall, Lincoln, NE 68583, USA; Complex Biosystems Interdisciplinary Life Sciences Program, Institute of Agriculture and Natural Resources, University of Nebraska-Lincoln, 2200 Vine Street, Lincoln, NE 68583, USA; Nebraska Center for Virology, University of Nebraska-Lincoln, 4240 Fair Street, Lincoln, NE 68583, USA; Department of Plant Pathology, University of Nebraska-Lincoln, 406 Plant Science Hall, Lincoln, NE 68583, USA; Nebraska Center for Virology, University of Nebraska-Lincoln, 4240 Fair Street, Lincoln, NE 68583, USA; Department of Plant Pathology, University of Nebraska-Lincoln, 406 Plant Science Hall, Lincoln, NE 68583, USA

**Keywords:** CP-RT, genome variation, genomic analysis, polerovirus, P0, VPg

## Abstract

The polerovirus (family *Solemoviridae*, genus *Polerovirus*) genome consists of single-, positive-strand RNA organized in overlapping open reading frames (ORFs) that, in addition to others, code for protein 0 (P0, a gene silencing suppressor), a coat protein (CP, ORF3), and a read-through domain (ORF5) that is fused to the CP to form a CP-read-through (RT) protein. The genus *Polerovirus* contains twenty-six virus species that infect a wide variety of plants from cereals to cucurbits, to peppers. Poleroviruses are transmitted by a wide range of aphid species in the genera *Rhopalosiphum, Stiobion, Aphis*, and *Myzus*. Aphid transmission is mediated both by the CP and by the CP-RT. In viruses, mutational robustness and structural flexibility are necessary for maintaining functionality in genetically diverse sets of host plants and vectors. Under this scenario, within a virus genome, mutations preferentially accumulate in areas that are determinants of host adaptation or vector transmission. In this study, we profiled genomic variation in poleroviruses. Consistent with their multifunctional nature, single-nucleotide variation and selection analyses showed that ORFs coding for P0 and the read-through domain within the CP-RT are the most variable and contain the highest frequency of sites under positive selection. An order/disorder analysis showed that protein P0 is not disordered. In contrast, proteins CP-RT and virus protein genome-linked (VPg) contain areas of disorder. Disorder is a property of multifunctional proteins with multiple interaction partners. The results described here suggest that using contrasting mechanisms, P0, VPg, and CP-RT mediate adaptation to host plants and to vectors and are contributors to the broad host and vector range of poleroviruses. Profiling genetic variation across the polerovirus genome has practical applications in diagnostics, breeding for resistance, and identification of susceptibility genes and contributes to our understanding of virus interactions with their host, vectors, and environment.

## 1. Introduction

**Figure 1.**
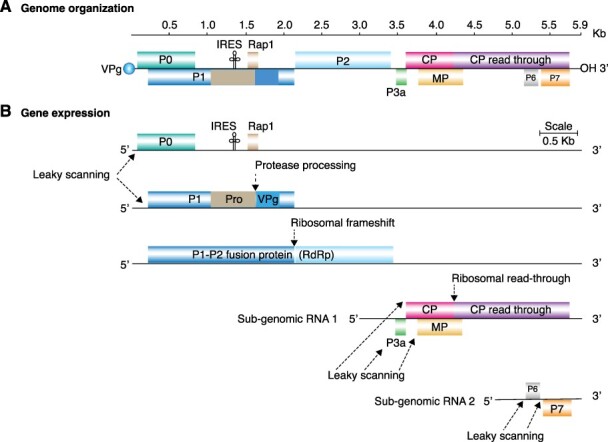
Representation of polerovirus genome organization and gene expression. Lines represent non-coding regions and labeled boxes represent ORFs. (A) Generalized polerovirus genome organization. Coordinates are based on PLRV accession number KY856831. (B) Gene expression strategies include the formation of two sub-genomic RNAs, translation by IRES-mediated internal initiation, leaky scanning, ribosomal frameshift, and ribosomal read-through. Protein 1 is processed into mature VPg by proteolysis. Pro, putative protease; Rap1, replication-associated protein; CP, capsid protein and read-through domain; p3a, protein essential for systemic virus movement; IRES, internal ribosomal entry site.

The plant virus family *Solemoviridae* consists of four genera: *Sobemovirus, Polemovirus, Enamovirus*, and *Polerovirus* (Walker et al. 2021). *Enamovirus* and *Polerovirus* were previously in the family *Luteoviridae* until they were added to the family *Solemoviridae* in 2021 (Walker et al. 2021). The genome consists of positive-, single-stranded RNA ranging from 5.6 to 6.2 kb (Table 1), protected by a virus protein genome-linked (VPg) cap at the 5ʹ untranslated region (UTR) and organized in five to six overlapping open reading frames (ORFs), some of which are translated from subgenomic RNAs (Krueger et al. 2013; Somera, Sarmiento, and Truve 2015). Diversification in the *Solemoviridae* initiated with a splitting event 900 years ago related to agricultural expansion (Pagan and Holmes 2010). The *Polerovirus* and *Enamovirus* genera are differentiated based on the nucleotide sequence and organization of their ORFs. The main difference is the lack of a movement protein in enamoviruses. Both poleroviruses and enamoviruses contain the RNA-dependent RNA polymerase (RdRp) that is a translational fusion of ORF1 (P1) through ORF2 (P2), a coat protein (CP, ORF3), and a read-through domain (ORF5; Fig. 1). The CP is essential for virion formation and is also involved in aphid transmission and virus movement (Kaplan et al. 2007; Smirnova et al. 2015). The CP-RT protein, formed by the translational fusion of ORF3 and ORF5, has been implicated in vector transmission and virus movement (Smirnova et al. 2015). In the process, rather than stopping at the end of ORF3, ribosomes incorporate one amino acid and continue to translate ORF5 (Xu et al. 2018). The read-through domain encoded by ORF5 is only expressed as the fusion protein CP-RT. Both the CP and the CP-RT are incorporated into the T = 3 icosahedral 23–25-nm virion (Boissinot et al. 2014; Xu et al. 2018).

**Table 1.** Polerovirus species downloaded from GenBank (November 2019). For each virus species, one accession was chosen as reference. The analysis described in this paper was based only on species, with at least three accessions with ≥95 per cent of the length of the reference accession and ≥90 per cent nucleotide identity with other accessions within the same virus species. For each species, the number of available accessions, the accession used as reference, the length of the reference, the 95 per cent length cutoff, and the number of accessions at least 95 per cent of the reference length are indicated.

Species	Total accessions	Reference	Length (nt)	95% length	Accessions (>95%)
*African eggplant yellowing virus* ^a^	4	KX856972	5953	5655	3
*Barley virus G* ^a^	13	NC_029906.1	5620	5339	6
*Beet chlorosis virus*	35	NC_002766.1	5777	5488	4
*Beet leaf yellowing virus* ^a^	4	LC428352.1	5670	5387	4
*Beet mild yellowing virus*	41	NC_003491.1	5723	5437	5
*Beet western yellows virus*	129	NC_004756.1	5744	5457	22
*Brassica yellows virus* ^a^	37	NC_016038.2	5678	5394	19
*Carrot red leaf virus*	28	NC_006265.1	5726	5440	6
*Cereal yellow dwarf virus-RPS*	4	NC_002198.2	5662	5379	4
*Cereal yellow dwarf virus-RPV*	83	NC_004751.1	5778	5489	5
*Cotton leafroll dwarf virus*	150	NC_014545.1	5866	5573	7
*Cucurbit aphidborne yellows virus*	667	KR231942.1	5683	5399	46
*Luffa aphidborne yellows virus* ^a^	12	NC_027703.1	5961	5663	4
*Maize yellow mosaic virus*	97	MK652150.1	5642	5360	45
*Melon aphidborne yellows virus*	18	NC_010809.1	5676	5392	3
*Pepo aphidborne yellows virus*	46	NC_030225.1	5813	5522	3
*Pepper vein yellows virus*	127	NC_015050.1	6244	5932	9
*Phasey bean mild yellows virus^a^*	5	KT962999.1	5838	5546	4
*Potato leafroll virus*	264	NC_001747.1	5987	5688	38
*Strawberry polerovirus-1* ^a^	15	NC_025435.1	5986	5687	4
*Suakwa aphidborne yellows virus* *^a^*	26	NC_018571.2	5845	5553	3
*Sugarcane yellow leaf virus*	522	NC_000874.1	5899	5604	47
*Turnip yellows virus*	87	NC_003743.1	5698	5413	16

^a^ Species described as unclassified poleroviruses in GenBank and not included in the most current list of species in the genus *Polerovirus* (Walker et al. 2021).

The distinctive feature of poleroviruses and enamoviruses is the presence of ORF0 encoding protein 0 (P0), which is a suppressor of gene silencing (Pazhouhandeh et al. 2006; Baumberger et al. 2007; Csorba et al. 2010). Poleroviruses contain P3a (ORF3a) for long-distance movement and a phloem-restricting, cell-to-cell movement protein at P4 (ORF4; Fig. 1). In contrast, enamoviruses lack P3a and P4 movement proteins (Silva et al. 2017). Additionally, poleroviruses encode P6 (ORF6) and P7 (ORF7). *Potato leafroll virus* (PLRV) P7 inhibits the aphid induction of ethylene and enhances aphid fecundity (Patton et al. 2020). The biological roles of P6 remain to be determined (Delfosse et al. 2021). P0, VPg, and CP contribute to vector specificity (Patton et al. 2020), while the read-through domain within CP-RT is important for vector transmission, virus movement, and accumulation (Peter et al. 2008).

Poleroviruses are a diverse genus with a broad host range. Currently, there are twenty-six official species (Walker et al. 2021) along with several unofficial species listed in GenBank with complete genome accessions (Table 1). Poleroviruses are distributed worldwide, and some species cause damaging diseases in a wide variety of plants including potato, sugarcane, maize, and beets (Garcia-Ruiz, Holste, and LaTourrette 2021). The type species for poleroviruses is PLRV (Taliansky, Mayo, and Barker 2003; Delfosse et al. 2021). Poleroviruses are obligatorily transmitted by aphids, and infection is limited to the phloem. As such, symptoms generally include stunting, yellowing, leaf malformations, and discoloration of the main leaf vein (Distéfano, Kresic, and Hopp 2010; Fiallo-Olive et al. 2018).

The polerovirus genome forms two subgenomic RNAs during replication, which are translated through several mechanisms (Fig. 1; Smirnova et al. 2015). By containing alternative initiation codons within ORF0, leaky scanning is used to translate P1. P1 is important for viral replication and can be expressed alone or fused with P2 when a ribosomal frameshift occurs (Prufer et al. 1992; Nixon et al. 2002; Nickel et al. 2008; Smirnova et al. 2015; Delfosse et al. 2021). In addition, VPg is released from P1 by proteolysis (Osman, Coutts, and Buck 2006). From subgenomic RNA 1, leaky scanning is used to translate P3a, putative movement protein (MP), and CP (Smirnova et al. 2015). P4 is a movement protein that enables both cell-to-cell movement through the plasmodesmata and systemic movement (Ju et al. 2017; Delfosse et al. 2021).

Viruses must retain flexibility in their genomes to adapt to different hosts and vectors (Rantalainen et al. 2011; Garcia-Ruiz 2018; Nigam and Garcia-Ruiz 2020). Virus variation, evolution, and host adaptation are genetically determined (Obenauer et al. 2006; Moury and Simon 2011; Nigam et al. 2019; LaTourrette et al. 2021). This evolutionary process is evident through the emergence of new virus strains or species with novel properties and is mediated by the preferential accumulation of mutations in particular areas of the genome (Obenauer et al. 2006; Moury and Simon 2011; Nigam et al. 2019; LaTourrette et al. 2021). Further, in poleroviruses, RNA recombination is frequent and contributes to the emergence of new species or strains (Dombrovsky et al. 2013; Ibaba, Laing, and Gubba 2017). The most common cross-over sites occur in areas coding for the RdRp, VPg, and the CP and in the non-coding intergenic region between ORF2 and ORF3 (Pagan and Holmes 2010; Dombrovsky et al. 2013; Ndikumana et al. 2017; Kwak et al. 2018), which is the 5ʹ UTR of subgenomic RNA 1 (Miller, Dinesh-Kumar, and Paul 1995).

In poleroviruses, mutations have been mapped for some species or specific proteins. An analysis of the genome of nine polerovirus species showed that single-nucleotide polymorphisms (SNPs) were concentrated on ORFs at the 5ʹ end (P0 and P1) and 3ʹ end (CP-RT) and were lower between ORFs P2 through P4 (Huang et al. 2005). Within the P1–P2 fusion that forms the RdRp, the P2 portion is conserved (Koonin and Dolja 1993; Delfosse et al. 2021). Consistent with these observations, in *Rice yellow mottle virus* (RYMV, genus *Sobemovirus*), the VPg, located near the 5ʹ end, is hypervariable and mediates the emergence of resistance breaking strains (Hebrard et al. 2010, 2018).

Since the polerovirus genome contains overlapping ORFs (Smirnova et al. 2015), mutations have the potential to affect multiple proteins (Fig. 1). However, a comprehensive profile of variation in the polerovirus genome is not currently available. Here, we used SNPs, nucleotide diversity, and selection analyses to measure and characterize accumulation of mutations in the genome of poleroviruses. Results showed that variation patterns are conserved across species in the genus *Polerovirus*: the most genetically stable ORF codes for RdRp. In contrast, ORFs coding for P0 and CP-RT, specifically the read-through domain, are hypervariable and have the highest number of sites under positive and negative selection. Furthermore, the N-terminal part of the read-through domain, which is involved in aphid transmission (Peter et al. 2008), is genetically stable and ordered. In contrast, the C-terminal half is variable and highly disordered. Similarly, polerovirus VPg protein is variable and disordered. These features suggest that proteins P0 and VPg are determinants of host adaptation and point to CP-RT as a determinant of both host adaptation and vector transmission. These findings point to areas to target for future studies involving universal polerovirus diagnostic tests, breeding for virus resistance, and identification of susceptibility genes.

## 2. Materials and methods

All computational analyses were conducted using the high-performance computing nodes at the University of Nebraska-Lincoln Holland Computing Center. In-house bash and python scripts developed for this study are available upon request.

### 2.1 Genomic RNA sequences

Genomic sequences for all polerovirus species were downloaded from NCBI on 14 November 2019 using customized scripts based on Entrez Programming Utilities. One accession for each species was chosen as the reference genome. This accession was either the NCBI-designated reference accession for the species, or, if NCBI did not have a designated reference genome, the accession with the longest sequence was chosen (Table 1). The reference genome was used to determine the coordinates for each ORF. From the downloaded accessions, all accessions with <95 per cent the length of the reference genome were removed to ensure only almost complete or complete sequences were analyzed. All sequences with >2.5 per cent of unknown characters were also removed. For the accessions that passed through these filters, each accession was then compared to similar sequences using the NCBI Basic Local Alignment Search Tool server. Since sequences can be mislabeled, this served as a method to ensure that each accession would be analyzed as the correct species to avoid poor alignments due to large sequence dissimilarity. Any accession that showed the highest identity to a virus species outside of its labeled species was discarded. Further, all accessions that showed less than a 90 per cent nucleotide identity with other accessions within a virus species were discarded. Finally, any accessions that remained in question were compared directly to the reference accession and were discarded if they had less than a 90 per cent nucleotide identity. For variation analyses, only species with at least three accessions were used to ensure meaningful statistical comparisons (Table 1; Shen et al. 2010).

### 2.2 Phylogenetic tree

**Figure 2.**
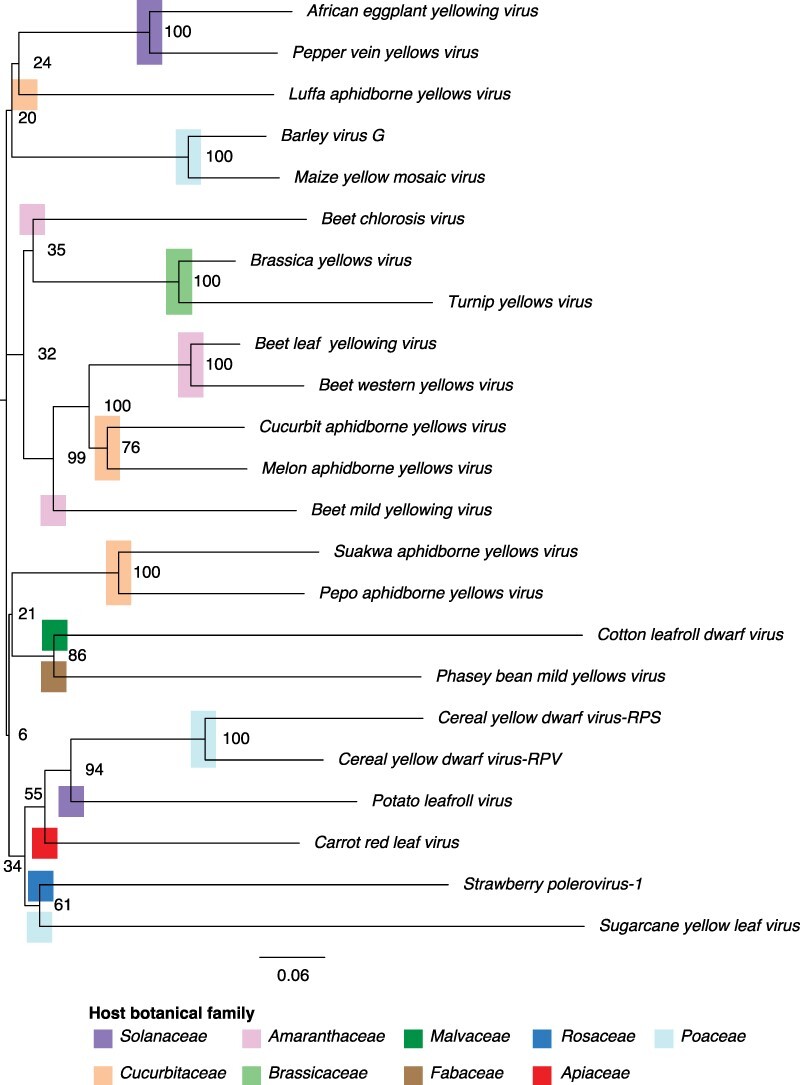
Polerovirus phylogeny based on consensus nucleotide sequences. Neighbor-joining tree with bootstrap values (100) generated using MAFFT. Plant host family is marked by colored bars.

All species with at least three accessions remaining after filtering were included in the phylogenetic tree (Fig. 2). Consensus sequences were derived for each species using custom scripts. Consensus sequences were combined and aligned using MAFFT version 7.4 (Multiple Alignment using Fast Fourier Transform) to form a neighbor-joining tree. Newick files of this alignment were transferred to Figtree version 1.4.3. for visualization (Rambaut 2009).

### 2.3 Genomic diversity

For all poleroviruses, alignment files (.aln) based on forward reads from MAFFT were downloaded and analyzed for SNPs and nucleotide diversity (Pi) in a 50-nt window as described (Nigam et al. 2019). Nucleotide diversity was analyzed using *Tassel version 5.0* (Bradbury et al. 2007). Pi, rather than SNPs, was used to determine the five most variable poleroviruses as it normalizes for the number of accessions. For both SNPs and Pi, the average and 99 per cent confidence interval was estimated (*P*-value <0.01; Hazra 2017). SNPs and Pi were mapped across the genome for the five most variable poleroviruses and PLRV.

### 2.4 Selection analysis

Positive and negative selection sites were identified for each ORF for the five most variable poleroviruses and PLRV. For each ORF, sequences were obtained using custom python scripts. To obtain P1–P2 coding sequence, the frameshift nucleotide was deleted to allow for P1–P2 translation. For the CP-RT, the CP stop codon was changed from UAG to CAG to allow for translation. Sequences were translated using *EMBOSS Transeq* online tool (https://www.ebi.ac.uk/Tools/st/emboss_transeq/), last accessed on 20 November 2021 (Madeira et al. 2019). Sequences were aligned using MAFFT and alignment files inputted into single-likelihood ancestor counting (SLAC) and MEME tools at http://www.datamonkey.org/ last accessed on 20 November 2021. A significance level ≤0.05 and >0.95 posterior probability was used for both SLAC and MEME (Murrell et al. 2012). When normalizing to the length of the ORF, for the P1–P2 fusion protein and the CP-RT, sites were counted only for the sections of protein from frameshift and read-through onward, respectively. P3a was normalized to the length of the window.

### 2.5 P0 and CP-RT annotated phylograms

For selected viruses, a phylogram was generated based on either available P0 or CP-RT protein sequences using MAFFT version 7.4 to form a neighbor-joining tree. Geographical location and host were annotated for each sequence using GraPhlan (Asnicar et al. 2015; Nigam et al. 2019).

### 2.6 Protein disorder

Disorder and order were mapped for P0 and the CP-RT polyproteins using the Protein DisOrder prediction System (PrDOS; Ishida and Kinoshita 2007). PrDOS predicts disorder based on a sliding window analysis of the amino acid sequence combined with disorder of template or homologous proteins. The reference accession for the selected viruses was used as inputs. For measuring VPg disorder, accessions on GenBank did not differentiate P1 and VPg coordinates, so UniProt accessions P11622 for PLRV and P09506 for *Turnip yellows virus* (TuYV) were used instead. These accessions were used as the VPg region was specifically differentiated from the rest of the P1 protein. Disorder and order were mapped using the Multilayered Fusion-based Disorder predictor (MFDp). MFDp is a meta-predictor composed of several different disorder predictors, primarily DISOPRED, DISOclust, IUPRED-S, and IUPRED-L (Mizianty et al. 2010). For both disorder predictors, regions were colored based on predicted order and disorder and plotted by their disorder probability. The threshold of 0.5 represents a false positive rate of 5 per cent.

## 3. Results

### 3.1 Polerovirus phylogeny is related to the botanical family of their hosts

To determine the genetic relationships across all polerovirus species, a nucleotide-based phylogenetic tree was created using the twenty-three polerovirus species for which at least three accessions remained after quality control filtering (Fig. 2). Poleroviruses formed a monophyletic group with several clusters not related to the botanical family of the hosts. The two poleroviruses that infect brassicaseas clustered near each other. Out of the three poleroviruses that infect solanaceas, two formed a cluster. PLRV grouped next to viruses infecting plants in the *Poaceae* and away from viruses infecting *Solanaceae.* The five poleroviruses that infect cucurbits grouped in three clusters, and those that infect grasses formed two clusters (Fig. 2). *Cotton leafroll dwarf virus* and *Phasey bean mild yellows virus* clustered close to species infecting cucurbits. These results suggest that sequence similarity between polerovirus species does not correlate with the botanical family of their host. It is possible that poleroviruses face selection pressure from their vectors and that this clustering is influenced by vector specificity.

### 3.2 Genome-wide nucleotide variation

To measure nucleotide variation along the entire genome, for the twenty-three polerovirus species with at least three different accessions, we used SNPs and nucleotide diversity (Nigam et al. 2019).

**Figure 3.**
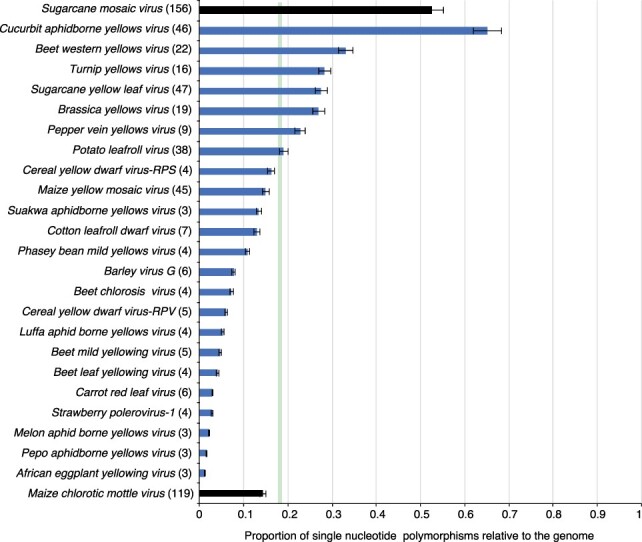
SNPs in poleroviruses. For each species and for the entire genome, SNPs were estimated in a 50-nt window. Bars represent the average (and standard error) proportion of polymorphic sites (number of SNPs/length of the genome). For each species, the number of nucleotide accessions is indicated in parenthesis. The green vertical line represents the mean and a 99 per cent confidence interval (*P*-value <0.01). For comparison, SCMV and MCMV were used as hypervariable and genetically stable, respectively.

*Cucurbit aphidborne yellows virus* (CABYV) and *Beet western yellows virus* (BWYV) had the highest accumulation of nucleotide substitutions with at least 30 per cent of their genome being polymorphic. This is similar to the variation observed for *Sugarcane mosaic virus* (SCMV), a highly variable potyvirus (Nigam et al. 2019) used as a control in the analysis (Fig. 3). The mean for the entire *Polerovirus* genus was similar to the variation observed for *Maize chlorotic mottle virus* (MCMV; Fig. 3), an RNA virus with low genetic diversity (Braidwood et al. 2018). In nine of the twenty-three poleroviruses, genomic variation was at least 15 per cent. This is higher than that observed for MCMV. In fourteen of the twenty-three poleroviruses, genomic variation was less than that observed for MCMV (Fig. 3).

**Figure 4.**
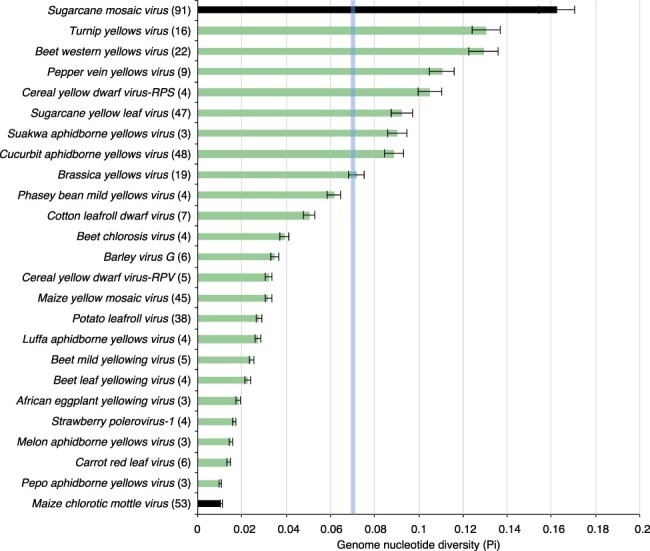
Nucleotide diversity (Pi) in all poleroviruses with three or more accessions. Bars represent the proportion of variable positions with respect to the length of the genome and normalized to the number of accessions. Labels are as in Fig. 3.

To account for differences in the number of accessions, variation was measured using a nucleotide diversity index (Pi) that normalizes variation to the number of accessions (Nigam et al. 2019). In twenty-two out of twenty-three poleroviruses, the nucleotide diversity was higher than that observed for MCMV (Fig. 4). These results show that poleroviruses are highly variable, with some species accumulating mutations to levels that are similar to those observed in potyviruses such as SCMV (Nigam et al. 2019).

Based on the nucleotide diversity, TuYV, BWYV, *Pepper vein yellows virus* (PVYV), *Cereal yellow dwarf virus-RPS* (CYDV-RPS), and *Sugarcane yellow leaf virus* (SCYLV) had the highest variation (Fig. 4) and, along with the type species, PLRV, were used for all further downstream analyses.

### 3.3 Nucleotide diversity and selection by ORF

**Figure 5.**
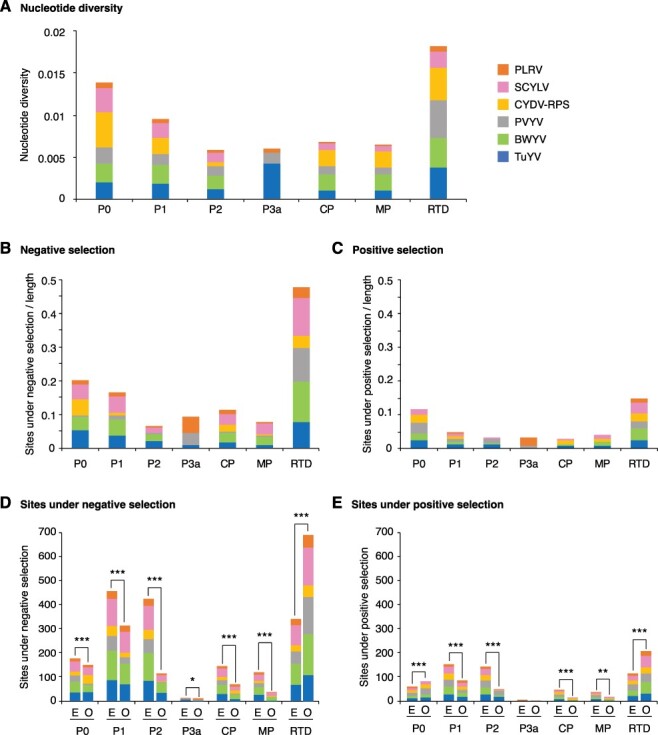
Nucleotide diversity, positive, and negative selection in the top five most variable poleroviruses and PLRV. Parameters were estimated by ORF and normalized to the corresponding length. Virus names are color-coded. (A) Cumulative nucleotide diversity by virus and ORF. (B) Sites under negative selection expressed as a proportion with respect to the length of the ORF. (C) Sites under positive selection expressed as a proportion with respect to the length of the ORF. (D) Expected (E) and observed (O) number of sites under negative selection sites. Expected values were determined assuming random distribution in the genome. (E) Expected and observed number of sites under positive selection. The * denotes significant differences with *P*-value ≤0.05, ** for *P*-value ≤0.01, and *** for *P*-value ≤0.001 as calculated by the chi-squared test.

For the five most variable poleroviruses and PLRV, nucleotide diversity was measured per ORF. The read-through domain within the CP-RT showed the highest nucleotide diversity followed by P0 and P1. In contrast, CP, MP, P3a, and P2 showed the lowest nucleotide diversity (Fig. 5A). Using SLAC and MEME, negative (Fig. 5B) and positive (Fig. 5C) selection sites were mapped and normalized across each ORF. The abundance of negative selection sites across an ORF was 1.78- to 4.38-fold higher than positive selection sites, showing that polerovirus genomes are primarily under negative selection. After normalizing sites under negative selection to the length of the ORF, the read-through domain had the highest frequency of sites under negative selection followed by P0 and P1 (Fig. 5B). The read-through domain and P0 have the highest frequency of sites under positive selection (dN/dS ratio >1, *P* ≤ 0.05; Fig. 5C). Next, the number of negative and positive selection sites observed was compared to the number expected in each ORF if mutations were randomly distributed. Only the read-through domain had a higher number of negative selection sites than would be expected randomly. In all other ORFs, the observed number of sites under negative selection was lower than would be expected randomly (Fig. 5D). P0 and the read-through domain had a higher number of sites under positive selection sites than expected randomly (Fig. 5E). Accordingly, the most variable ORFs (the read through domain and the PO) also had the highest number of sites under both negative and positive selection.

These results show that, in poleroviruses, P2 is one of the most genetically stable ORFs with low nucleotide diversity, a high number of negative selection sites, and a low number of positive selection sites. In contrast, the read-through domain and P0 contain hypervariable areas with a high number of both positive and negative selection sites.

### 3.4 ORFs coding for P0 and the read-through domain are the most variable

**Figure 6.**
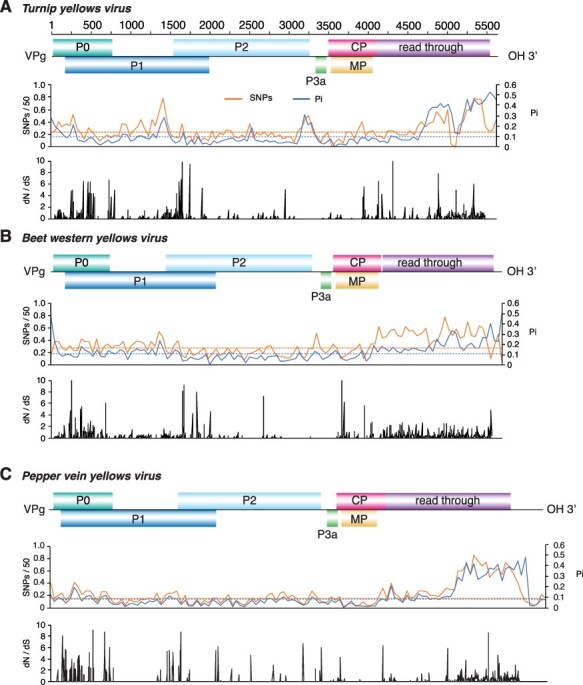
Genome-wide variation in selected poleroviruses. SNP and nucleotide diversity (Pi) and the ratio of non-synonymous to synonymous changes (dN/dS) were estimated in 50-nt window. The average and a 99 per cent confidence interval (*P*-value <0.01) is indicated as a horizontal line. ORFs are color-coded and labeled with the name of the protein they encode. (A) TuYV. Coordinates are based on accession NC_003743.1. (B) *Beet western yellows virus*. Coordinates are based on accession NC_004756.1. (C) PVYV. Coordinates are based on accession NC_015050.1.

**Figure 7.**
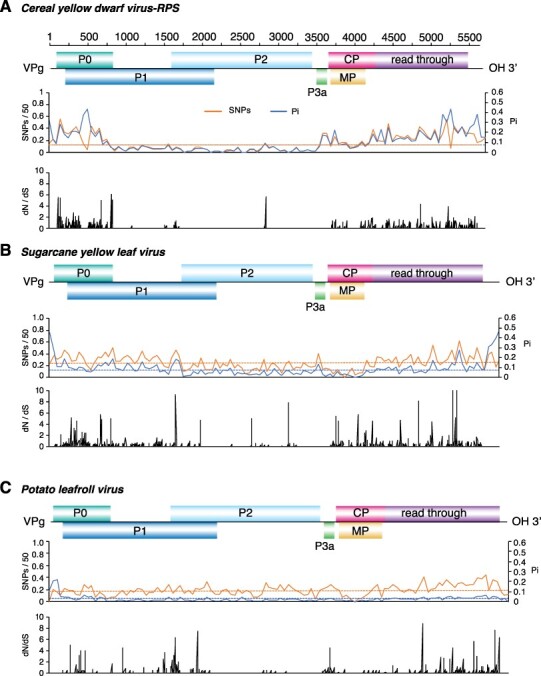
Genome-wide variation of three poleroviruses. Labels are as in Fig. 6. (A) CYDV-RPS. Coordinates are based on accession NC_002198.2. (B) SCYLV. Coordinates are based on accession NC_000874.1 (C) PLRV. Coordinates are based on accession NC_001747.1.

Results described above showed that accumulation of mutations in the polerovirus genome is not random. Instead, mutations preferentially accumulate in ORFs coding for P0 and the read-trough domain within CP-RT. To further characterize the distribution of mutations, using the most variable species and PLRV, SNPs and nucleotide diversity were estimated in a 50-nt window and normalized. For each species, a map was generated by plotting SNPs and nucleotide diversity against the genome to create an identity plot. Genome-wide maps confirmed that nucleotide variation is not distributed randomly. Instead, mutations preferentially accumulated in the ORFs coding for P0, the read-through domain of the CP-RT, and in the intergenic region between P2 and P3a. All six poleroviruses showed a low variation in ORF2 coding for P2, confirming that this is the most genetically stable genome area (Figs 6 and 7).

In viruses, areas of the genome under positive selection have flexibility related to an expanded host range (Bedhomme, Lafforgue, and Elena 2012). To identify areas under positive or negative selection in poleroviruses, we used SLAC and MEME on individual ORFs of the five most variable poleroviruses and PLRV. Results showed that areas containing sites under positive selection (dN/dS ratio > 1, *P* value ≤0.05) mapped across P0 and the C terminal part of the read-through domain. Additionally, areas under positive selection were detected at the CP and MP overlap. P2 and P3a had the least sites under positive selection. The distribution of sites under negative selection followed the same pattern with the most occurring at P0 and the read-through domain portion of the CP-RT and the least in P2 and P3a (Figs 6 and 7).

These results show that, in poleroviruses, mutations preferentially accumulate in ORFs coding for P0, the read-through domain within the CP-RT, and in the intergenic region between P2 and P3a region. P0 and the read-through domain also contain the highest frequency of sites under negative and positive selection (Figs 6 and 7), suggesting that they are important for host adaptation, vector adaptation, or both.

### 3.5 The read-through domain within CP-RT protein contains disordered areas

**Figure 8.**
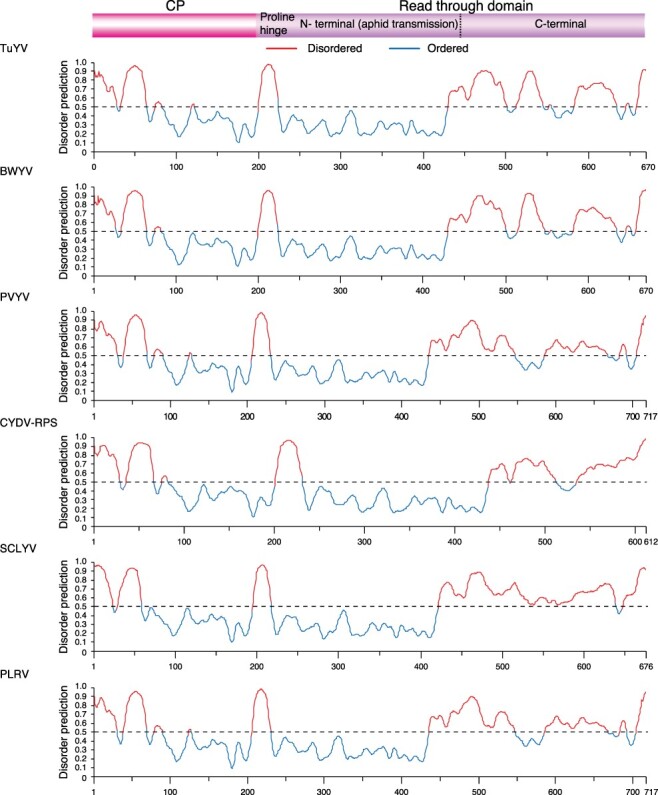
Disorder of CP-RT of the top five most variable polerovirus and type species. Disorder across CP-RT mapped using PrDOS with disorder predictions color-coded above and below 0.5 representing disorder and order, respectively.

Intrinsically disordered proteins and intrinsically disordered protein regions are often associated with protein–protein interactions between multiple or diverse interaction partners, and they regulate important processes such as transcription, translation, and assembly of protein complexes (Uversky 2002; Szilagyi, Gyorffy, and Zavodszky 2008). For each of the five most variable poleroviruses and PLRV, the disorder of CP-RT was measured using PrDOS (Ishida and Kinoshita 2007). PrDOS averages disorder predictions using a support vector machine algorithm based on amino acid content and disorder of homologous proteins. The map identified areas of disorder and order at homologous locations in all poleroviruses analyzed. The N-terminus of the CP shows a large area of disorder ranging from fifty-eight to sixty-nine amino acids containing a small area of order ranging from four to seven amino acids. The C-terminus of CP and the N-terminus of the read-through domain also show an area of disorder ranging from twenty-three to thirty amino acids that correspond to the proline hinge (Peter et al. 2008). While the N-terminal part is ordered, the C-terminus of the read-through domain is almost entirely disordered (Fig. 8). The CP-RT is on average 43.59 per cent disordered, indicating that these proteins may play a role in host and vector adaptation by binding to several host and virus factors.

### 3.6 Protein P0 is ordered

**Figure 9.**
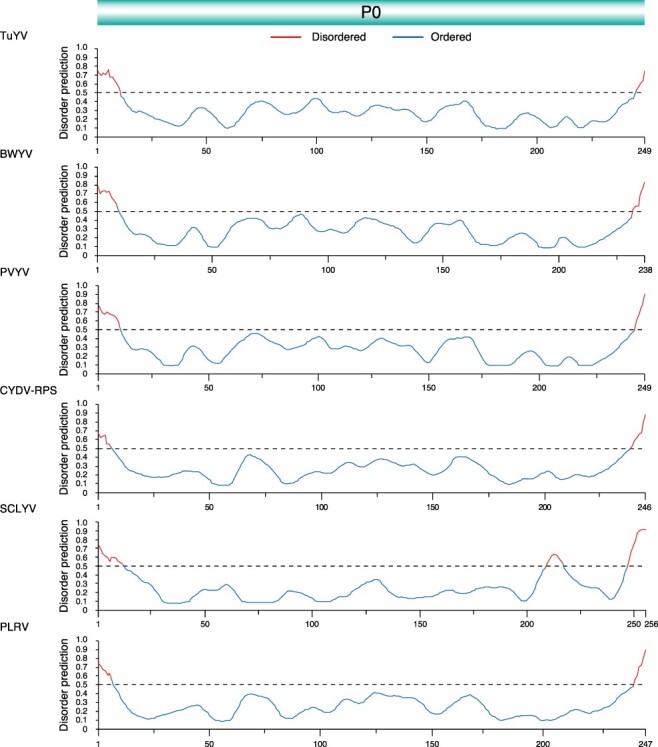
Disorder of P0 of the top five most variable polerovirus and type species. Disorder across P0 mapped using PrDOS with *P* = 0.05 threshold representing disorder and order, respectively. Colored based on PrDOS disorder and order prediction.

For all six poleroviruses analyzed, the only areas of disorder were at the start and end of the P0 protein with one additional area of disorder in SCYLV (Fig. 9). With an average 6.98 per cent disordered, P0 is a highly ordered protein. These observations suggest that hyper variation (Fig. 5) and disorder (Fig. 9) can be separated.

### 3.7 Contrasting evolutionary path between P0 and CP-RT

**Figure 10.**
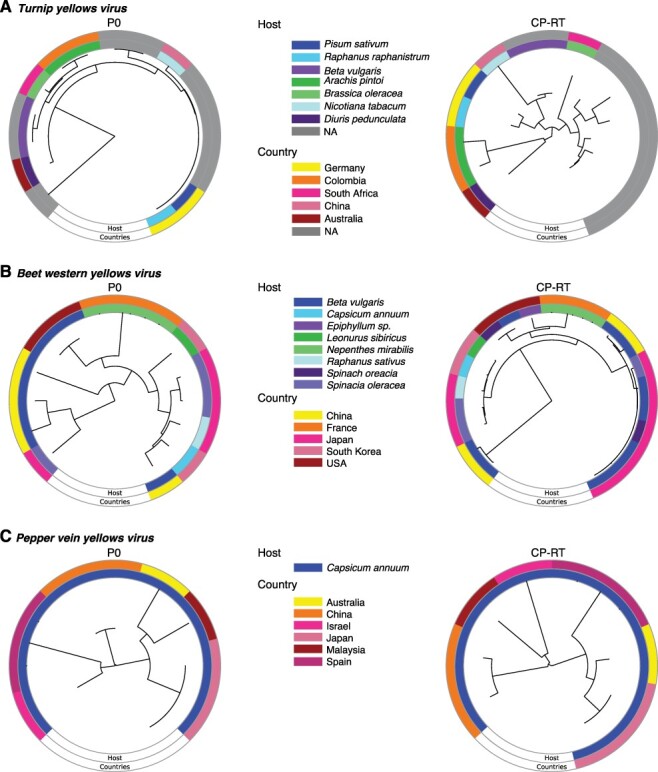
Phylogram based on P0 and CP-RT protein sequences. The neighbor-joining phylogenetic tree in the center was generated using MAFFT. Outer ring indicates country of origin and the inner ring the host. (A) TuYV. (B) *Beet western yellows virus*. (C) PVYV.

**Figure 11.**
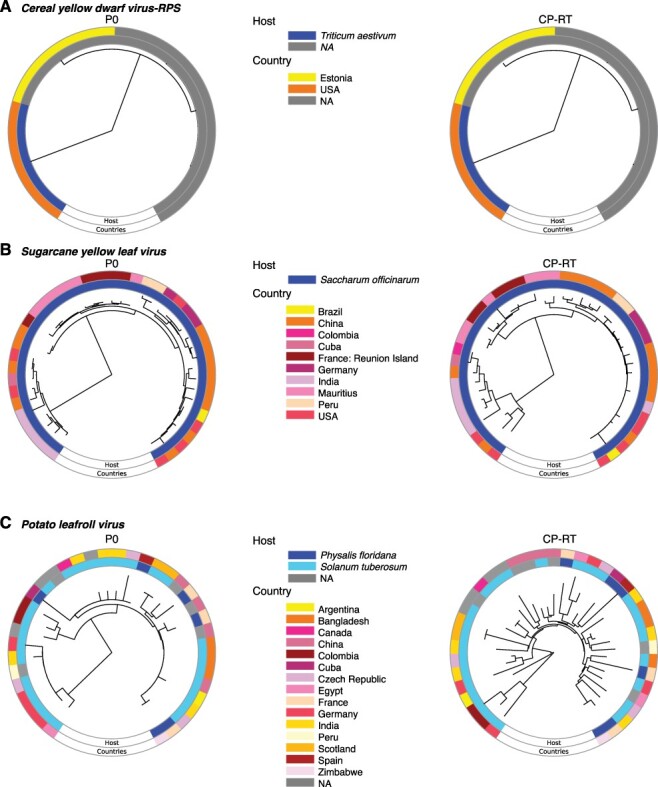
Phylogram based on P0 and CP-RT protein sequences. Labels are as in Fig. 10. (A) CYDV-RPS. (B) SCYLV. (C) PLRV.

To determine evolutionary patterns, phylogenies for each of the five most variable viruses and PLRV were created separately for P0 or CP-RT using protein sequences for each species. The host plant species and country of origin were added to the phylogeny. Except for CYDV-RPS, P0 and CP-RT resulted in phylogenetic trees with different structures (Figs 10 and 11). The CP-RT phylogenies had greater numbers of clades and branches, consistent with a higher accumulation of mutations in CP-RT than in P0 (Fig. 5). The viruses with the highest nucleotide diversity, TuYV and BWYV, had the highest number of host types, suggesting selection pressure to maintain flexibility is the key determinant of host adaptation. Accessions clustered together based on the host plant rather than by country of origin, emphasizing contribution of the host in fixing mutations within the virus population.

### 3.8 Polerovirus VPg is variable and disordered

VPg is covalently linked to the viral genome and acts as a cap at the 5ʹ UTR (Jiang and Laliberte 2011). VPg is a self-cleaving protease located near the C terminal part of P1 protein, is important for ribosome interactions, and is predicted to participate in viral RNA synthesis and translation (Osman, Coutts, and Buck 2006; Delfosse et al. 2021). Interactions between VPg and translation initiation factors are highly specific and determine compatibility between poleroviruses and their hosts (Hebrard, Pinel-Galzi, and Fargette 2008). This pattern can be seen in sobemoviruses as well (Jiang and Laliberte 2011). RYMV VPg interacts with rice translation initiation factor eIF(iso)4G1 encoded by the *RYMV1* gene. This highly specific interaction determines the compatibility between RYMV and rice (Hebrard, Pinel-Galzi, and Fargette 2008). Point mutations in *RYMV1* confer recessive resistance to RYMV by disrupting interaction between eIF(iso)4G1 and VPg. Interestingly, mutations in the VPg central domain restore interaction with eIF(iso)4G1 and break resistance (Hebrard, Pinel-Galzi, and Fargette 2008; Hebrard et al. 2010; Traore et al. 2010).

**Figure 12.**
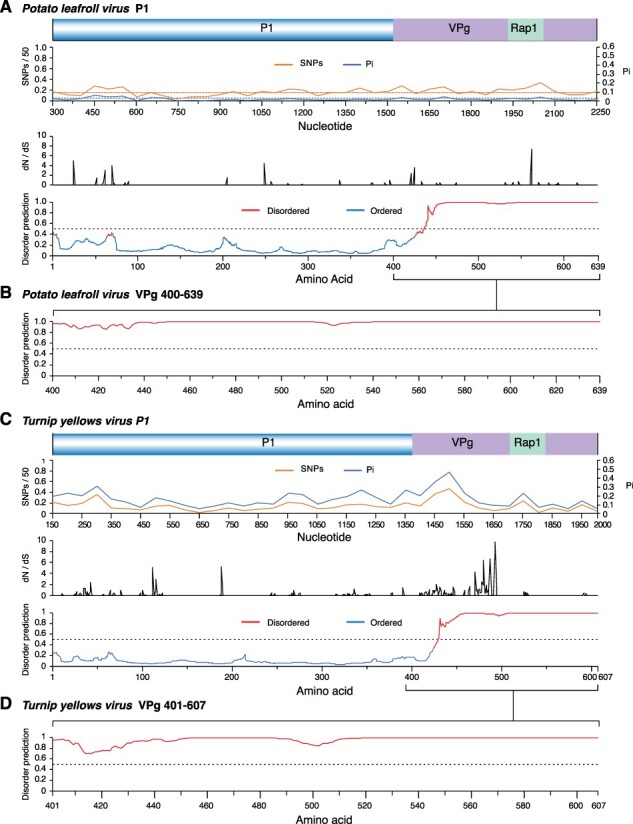
Nucleotide variation and order/disorder in PLRV and TuYV P1 and VPg. For nucleotide variation, labels are as in Fig. 5. Disorder was mapped using MFDp with *P* = 0.5 disorder threshold. (A) PLRV P1. Coordinates are based on accession NC_001747.1. (B) PLRV VPg. Sequence and coordinates are based on accession P11622. (C) TuYV P1. Coordinates are based on accession NC_003743.1. (D) TuYV VPg. Sequence and coordinates are based on accession P09506.

A high frequency of SNPs and nucleotide variation and a high number of positive and negative selection sites were detected at the end of the P1 protein (Figs 6, 7 and 12). The VPg domain is not annotated for most of the poleroviruses currently represented in GenBank. Only one annotated UniProt accession was available for VPg for both PLRV and TuYV. Thus, order/disorder was estimated for PLRV and TuYV P1, including VPg. The area that corresponds to VPg is disordered (Fig. 12A, C). To verify this result, we estimated order/disorder exclusively for VPg. The entire VPg is disordered for both viruses (Fig. 12B, D). These results show that polerovirus VPg is variable, contains sites under positive selection, is structurally flexible, and is intrinsically disordered.

## 4. Discussion

Poleroviruses are obligatorily transmitted by aphids (Kaplan et al. 2007) and during transmission and infection face evolutionary constraints imposed by factors in the insect vector, the host plant, the environment, and their interaction (Wan et al. 2015; Li et al. 2016; Nigam and Garcia-Ruiz 2020). When viruses and hosts co-evolve, interactions between host and virus factors determine compatibility or incompatibility (Garcia-Ruiz 2018). Viral factors have also been shown to affect the physiology of a host in order to support viral spread through vector feeding (Mauck, De Moraes, and Mescher 2014). Further, climate change is likely to increase the frequency of viral epidemics due to vector expansion into new geographical areas, thereby potentially exposing new hosts to the virus (Trebicki 2020). Since each host, vector, and environmental interaction has the chance to select against unfit viruses, viruses must maintain functionality and a high degree of fitness in order to remain in the population. Accordingly, poleroviruses must balance genomic flexibility and retaining essential functions.

Mutations in viral genomic RNA occur through nucleotide insertions, deletions, and substitutions, introduced randomly by viral RNA-dependent RNA polymerases during viral RNA replication (Garcia-Arenal, Fraile, and Malpica 2001). Additionally, RNA recombination allows for the rapid generation of genetic diversity, occurs during viral RNA replication, and requires the presence of two parental viral RNAs in a single replication compartment (Garcia-Ruiz, Diaz, and Ahlquist 2018). In poleroviruses, both intraspecific and interspecific RNA recombination events are frequent (Pagan and Holmes 2010). Mutations and new genomes formed through RNA recombination may have neutral, positive, or deleterious effects on virus fitness, leading to fixation or removal from the viral population (Moury and Simon 2011; Garcia-Ruiz, Diaz, and Ahlquist 2018; Nigam and Garcia-Ruiz 2020). New sequences with a positive effect may enable poleroviruses to infect new hosts and lead to the emergence of new strains or new species (Ibaba, Laing, and Gubba 2017). Under this model, accumulation of mutations in viral genomes is not random. Instead, mutations preferentially accumulate in areas that are key determinants of host adaptation, pathogenicity, and suppression of antiviral defense (Obenauer et al. 2006; Nigam et al. 2019; LaTourrette et al. 2021). This model predicts that within a virus genome, mutations preferentially accumulate in proteins that are determinants of host adaptation or vector transmission. The analyses described here showed preferential accumulation of mutations to particular areas of the polerovirus genome: ORF 0 and ORF 5, the silencing suppressor, and aphid transmission proteins.

The genome-wide variation profile described here was based on the twenty-three polerovirus species with three or more accessions in GenBank after filtering for quality (Table 1). Results showed that the *Polerovirus* genome is highly variable. Based on SNPs, CABYV and BWYV genomes were at least 30 per cent polymorphic (Fig. 3). This frequency of variation is similar or higher (Fig. 3) to that observed for the highly variable potyvirus SCMV (Nigam et al. 2019). Furthermore, in fourteen of the twenty-three poleroviruses, genomic variation was higher than that observed for MCMV (Fig. 3), an RNA virus with low genetic diversity (Braidwood et al. 2018). Based on Pi, twenty-two of the twenty-three poleroviruses were more variable than MCMV (Fig. 4). We used the five most variable poleroviruses and PLRV to characterize and map the distribution of mutations on the genome. ORFs with the highest accumulation of nucleotide substitutions were P0 and the read-through domain within the CP-RT (Fig. 5A). Mutations also accumulated to levels higher than the average of the genome in the intergenic region between P2 and CP (Figs 6 and 7). The ORF coding for P2 accumulated the lowest frequency of mutations. Viruses co-evolve with their hosts leading to selection pressure acting on both virus and host factors (Garcia-Arenal, Fraile, and Malpica 2001). Therefore, viruses must be able to successfully recognize and interact with heterogeneous host and vector factors (Obbard et al. 2006). Accordingly, hypervariable areas often mediate host adaptation, vector specificity, and virus evolution, as illustrated by RYMV (Traore et al. 2010; Hebrard et al. 2018).

Recombination generates genetic diversity by switching genetic segments of RNA between viral variants. Recombination is most common in positive-stranded RNA viruses and can result in new species, host expansion, or resistance-breaking strains (Nagy 2008; Traore et al. 2010; Garcia-Ruiz, Diaz, and Ahlquist 2018). Rather than occurring randomly in the genome, recombination occurs at specific hot spots in the genome. In poleroviruses, multiple recombination sites have been discovered between ORFs, primarily at the intergenic region between P2 and CP (Pagan and Holmes 2010; Dombrovsky et al. 2013; Kwak et al. 2018), which correlates with the transcription site of subgenomic RNA 1 (Miller, Dinesh-Kumar, and Paul 1995). Additional recombination spots are within the RdRp and the 5ʹ region of P1 (Pagan and Holmes 2010; Dombrovsky et al. 2013; Kwak et al. 2018). This suggests that the 5ʹ and 3ʹ halves of the genome could have different evolutionary histories due to recombination events, as indicated by the difference in phylogenetic trees between P0 and the CP-RT (Figs 10 and 11). Additionally, or alternatively, P0, the CP-RT, and the intergenic region, being hypervariable, tolerate mutations better than other parts of the genome; thus, recombination events within them are less likely to result in a fitness penalty.

P0 and the CP-RT had the highest number of sites under positive selection and the highest number of sites under negative selection. In both cases, the frequency was higher than expected randomly (Fig. 5). While the read-through domain within the CP-RT showed a high degree of disorder, particularly at the proline hinge and at the C-terminal half (Fig. 8), very little disorder was detected in P0 (Fig. 9). In contrast with the rest of P1, in PLRV and TuYV, VPg is variable and disordered and contains sites under positive selection (Fig. 12). VPg is intrinsically disordered in the genera *Potyvirus, Sobemovirus, Caliciviruses*, and *Nepovirus*, suggesting this may be a pattern across virus genera (Satheshkumar et al. 2005; Hebrard et al. 2009; Jiang and Laliberte 2011; Rantalainen et al. 2011; Charon et al. 2018).

In poleroviruses and sobemoviruses, VPg is 9‒13 kDa, multifunctional, caps viral RNA, and is involved in translation through specific interactions with translation initiation factors (Traore et al. 2010). Consistent with disorder predicted for poleroviral VPg, in sobemoviruses, VPg is disordered (Satheshkumar et al. 2005; Hebrard et al. 2009), lacks common motifs or domains, and has no sequence similarity (Satheshkumar et al. 2005; Hebrard et al. 2009). The only common feature is a WAD/WGD nucleotide triphosphate-binding motif (Tamm and Truve 2000; Hebrard et al. 2009). Structural disorder is consistent with the multifunctional property of VPg for poleroviruses (Osman, Coutts, and Buck 2006), sobemoviruses (Satheshkumar et al. 2005; Hebrard et al. 2009), caliciviruses (Hebrard et al. 2009), nepoviruses (Jiang and Laliberte 2011), and potyviruses (Jiang and Laliberte 2011). Disorder allows VPg to be functionally promiscuous and adapt its structure to a variety of required interactions (Rantalainen et al. 2008, 2011; Hebrard et al. 2010). VPg variants with higher measures of disorder are better able to restore infection because sequence and structural flexibility likely allow the virus to escape fitness penalties or to evolve mutations that overcome negative mutations (Traore et al. 2010; Charon et al. 2018; Hebrard et al. 2018). The structural flexibility of VPg is consistent with its multifunctional nature and explains the lack of amino acid sequence similarity across virus genera. These observations suggest polerovirus VPg is a contributor to plant host adaptation and has multiple roles, which remain to be determined. Understanding the link between intrinsic disorder in proteins and RNA virus adaptation could help better create methods for antiviral control.

Intrinsically disordered proteins and intrinsically disordered protein regions lack a fixed three-dimensional shape, giving them greater flexibility and plasticity. They often interact with multiple partners and are important for several essential biological functions, including transcription and signal transduction (Lieutaud et al. 2016). Disorder in areas that interact with host and vector proteins enables the infection of new hosts and provides mutational robustness to avoid deleterious effects from mutations (Xue et al. 2014). The disordered regions in CP-RT contain (Fig. 8) domains needed for aphid transmission, virus systemic movement, and virion formation (Peter et al. 2008). Disorder in polerovirus CP-RT explains previous observations on the host-dependent effect of mutations in the PLRV CP-RT (Peter et al. 2008).

In several poleroviruses, P0 is a gene silencing suppressor that targets AGO1 for degradation (Pazhouhandeh et al. 2006; Baumberger et al. 2007; Csorba et al. 2010). An F-box motif in P0 is required for specific binding with the host S-phase kinase-associated protein 1 (SKP1) proteins that lead to the downstream degradation of AGO1 (Li et al. 2019). The lack of disorder in P0 suggests that, despite mutational robustness, the structure of P0 must be maintained to preserve functionality in a genetically diverse set of host plants. Alternatively, or in addition, P0 interacts with host proteins that maintain a highly conserved structure. In support of this model, structure and function of AGO proteins are highly conserved in plants (Carbonell and Carrington 2015; Wu et al. 2015; Brosseau et al. 2020). In contrast, disorder in CP-RT and VPg suggests that they have multiple interaction partners potentially including both plant and vector factors. These factors may be the genetic determinants of susceptibility by participating in virus replication and/or movement (Garcia-Ruiz 2018).

The results described here suggest that, using contrasting mechanisms, P0, CP-RT, VPg, and the intergenic region mediate adaptation to host plants and to vectors and are contributors to the broad host and vector range of poleroviruses. Additionally, variation profiles described here established the basis for polerovirus diagnostics, breeding for polerovirus resistance, and identification of susceptibility genes to poleroviruses.
